# Experiences of ICU survivors in a low middle income country- a multicenter study

**DOI:** 10.1186/s12871-018-0494-8

**Published:** 2018-03-21

**Authors:** Lalitha Pieris, Ponsuge Chathurani Sigera, Ambepitiyawaduge Pubudu De Silva, Sithum Munasinghe, Aasiyah Rashan, Priyantha Lakmini Athapattu, Kosala Saroj Amarasiri Jayasinghe, Kerstein Samarasinghe, Abi Beane, Arjen M. Dondorp, Rashan Haniffa

**Affiliations:** 1 Sri Lanka Nursing Council, Colombo, Sri Lanka; 2 Network for Improving Critical care Systems and Training, Colombo, Sri Lanka; 3 National Intensive Care Surveillance, Ministry of Health, Quality Secretariat Building, Castle Street Hospital for Women, Colombo, Sri Lanka; 4 Intensive Care National Audit and Research Centre, London, UK; 5 Office of Director Medical Services, Ministry of Health, Colombo, Sri Lanka; 6 Faculty of Medicine, University of Colombo, Colombo, Sri Lanka; 7 Department of Health Sciences, Kristianstad University, Kristianstad, Sweden; 8 Mahidol Oxford Tropical Medicine Research Unit (MORU), Bangkok, Thailand; 9 University of Oxford, Oxford, UK

**Keywords:** Critical care, ICU experience, Stressors, Low middle income countries

## Abstract

**Background:**

Stressful patient experiences during the intensive care unit (ICU) stay is associated with reduced satisfaction in High Income Countries (HICs) but has not been explored in Lower and Middle Income Countries (LMICs). This study describes the recalled experiences, stress and satisfaction as perceived by survivors of ICUs in a LMIC.

**Methods:**

This follow-up study was carried out in 32 state ICUs in Sri Lanka between July and December 2015.ICU survivors’ experiences, stress factors encountered and level of satisfaction were collected 30 days after ICU discharge by a telephone questionnaire adapted from Granja and Wright.

**Results:**

Of 1665 eligible ICU survivors, 23.3% died after ICU discharge, 49.1% were uncontactable and 438 (26.3%) patients were included in the study. Whilst 78.1% (*n* = 349) of patients remembered their admission to the hospital, only 42.3% (*n* = 189) could recall their admission to the ICU. The most frequently reported stressful experiences were: being bedridden (34.2%), pain (34.0%), general discomfort (31.7%), daily needle punctures (32.9%), family worries (33.6%), fear of dying and uncertainty in the future (25.8%).

The majority of patients (376, 84.12%) found the atmosphere of the ICU to be friendly and calm. Overall, the patients found the level of health care received in the ICU to be “very satisfactory” (93.8%, *n* = 411) with none of the survivors stating they were either “dissatisfied” or “very dissatisfied”.

**Conclusion:**

In common with HIC, survivors were very satisfied with their ICU care. In contrast to HIC settings, specific ICU experiences were frequently not recalled, but those remembered were reported as relatively stress-free. Stressful experiences, in common with HIC, were most frequently related to uncertainty about the future, dependency, family, and economic concerns.

**Electronic supplementary material:**

The online version of this article (10.1186/s12871-018-0494-8) contains supplementary material, which is available to authorized users.

## Background

The availability of Intensive Care Units (ICUs) in Low Middle Income Countries (LMICs) is increasing; however information regarding patient experience including stress, and psychosocial impact for critical care survivors is very limited [1]. Admission to the ICU is often preceded by traumatic events leading to limited patient recall of their admission and ICU stay [2]. In common with many other LMIC settings, where competition for state critical care resources is fierce, in Sri Lanka (anecdotally) only those surgical patients with complications or perceived to have the highest risk for complications are admitted to ICUs.

Patients who experience critical illness are at their most vulnerable- physically, mentally and emotionally- during their ICU stay, where both the processes of critical illness and life saving interventions often result in loss of independence in the most basic activities; speaking, washing and feeding [3, 4]. Day-to-day ICU procedures such as tracheal suctioning, invasive line placement and repositioning are associated with acute pain and discomfort, resulting in anxiety and sensory hypersensitivity. Prolonged exposure and lengthy ICU stay have a positive correlation with neurological pain and muscle fatigue, requiring complex chronic pain management and physical rehabilitation. Similarly, the ICU environment, which often includes relentless 24-h activity results in loss of day-night differentiation, sleep disturbance and disorientation, and adds additional stressors to patients who are already both physically and emotionally burdened [2, 4, 5]. These experiences can lead to cognitive impairment, depression, and for some patients, post-traumatic stress disorder (PTSD) [3, 6, 7]. These long term effects impede patient recovery and return to normal life.

Stressful patient experiences during the ICU stay is associated with reduced satisfaction with the critical care experience [8]. Increasingly, measures to describe and evaluate the quality and outcome of critical care include patients’ psycho-social wellbeing and recovery alongside their physical recovery [9, 10]. Being satisfied with health care is important for patient wellbeing and public acceptance and is an important measure of the adequacy of healthcare services [11, 12]. Importantly, patients and members of the public are active stakeholders in ICU care initiatives, including support networks for survivors and quality improvement initiatives within the HIC setting [13].

Critical care in developing countries is a growing specialty. However, research regarding patient experiences comes almost exclusively from HIC settings. Patient experience, satisfaction, and psychosocial needs during recovery are likely to be influenced by social and economic factors such as education, wealth and societal and familial structures, which vary greatly between economic and cultural settings in addition to their reason for ICU admission. For example, in Sri Lanka - a lower middle income country with 2.59 ICU beds per 100,000 population where ICU care is free at the point of delivery- the majority of critical care admissions are emergencies, which provide limited opportunity for gathering pre-admission information [14]. Greater understanding of ICU survivor experiences in LMIC settings would provide information for future service planning, evaluation of current treatments and provide advocacy for patients.

This study, utilizing a critical care network established in a lower middle income country, describes the recall of ICU survivors’ experiences, stressors and their satisfaction with ICU services in Sri Lanka. The study also evaluates the association between ICU patient’s experiences and satisfaction of care, and reports patients’ suggestions for future service improvement.

## Methods

All consecutive adult patients (> 18 years) who were admitted to 32 state ICUs (19 mixed general, 6 medical and 7 surgical ICU’s) and who survived to ICU discharge were included [2]. The 32 states’ hospitals were selected to be representative of all provinces and state hospital categories within the country.

Name, age, gender, telephone number, length of ICU stay, discharge status, severity of illness (APACHE II score) and primary reason for ICU admission, using APACHE II diagnosis were obtained from the critical care registry - where data is input by clinical staff on the ICU.

Eligible patients were invited to participate in a telephone interview conducted by the first author one month after ICU discharge. Patients who were unable to communicate over the telephone at first contact were re-contacted one week later. If the patient was still unable to communicate at this time, they were excluded from the study. Verbal consent was obtained [15].Written consent was not sought from participants as contact details were extracted from the critical care registry and face-to-face contact with patients was not possible. All subsequent data collection was via telephone and written consent would not have been feasible in this setting. Participants were given the right to withdraw from the study at any time.

ICU experiences, stress and satisfaction were gathered using a telephone questionnaire (Additional file 1) adapted from two previous studies [2, 16]. The tool from Granja et al. has been previously successfully used in an Asian higher-middle income country setting [17]. Questions related to satisfaction with ICU care, stressful experiences, memories retained by the patient, the ICU environment, interaction with healthcare professionals, dreams, nightmares, sleep disturbances and difficulties in concentrating were included. Impact on quality of life for patients’ families and the associated burden of having a loved one in critical care were not explored. Questions pertaining to direct experience of aspects of care were measured on a five-point Likert scale, as proposed in the original studies; where 0 = ‘I don’t remember’, 1 = ‘It was not hard’, 2 = ‘It was indifferent’, 3 = ‘It was hard’, 4 = ‘It was very hard’ and 5 = ‘It was awful’ [2]. Patient satisfaction with the different aspects of care were also measured on a Likert scale where 1 = Excellent, 2 = Very good, 3 = Good, 4 = Fair, 5 = Poor and 6 = Not Applicable [16].

Responses to the survey questions were reported in the format “number who selected response (percentage)”. Missing responses were not imputed. Following completion of the pre-specified questions, all patients were invited to provide comments including suggestions on how their ICU experience could have been improved. All free text responses were coded by an investigator (CS) and frequencies calculated, with infrequent responses (< 5) being listed under “other”. Software package Stata 13 was used for analysis. Patient characteristics were compared between those who recalled ICU admission and those who did not using Pearson’s chi square test for categorical and Mann Whitney test for continuous variables [18, 19]. Normality testing on continuous variables was performed using the Shapiro-Wilks test [20]. A *p*-value of < 0.05 was taken to indicate significant non-normality in the variable. Normal variables were reported as mean (sd) and non-normal variables were reported as median (IQR).

Ethical approval was obtained from the Ethics Review Committee of the Faculty of Medicine, University of Colombo (Annex). Authorization was also obtained from the national intensive care registry working committee and Ministry of Health, Sri Lanka.

## Results

**Fig. 1:**
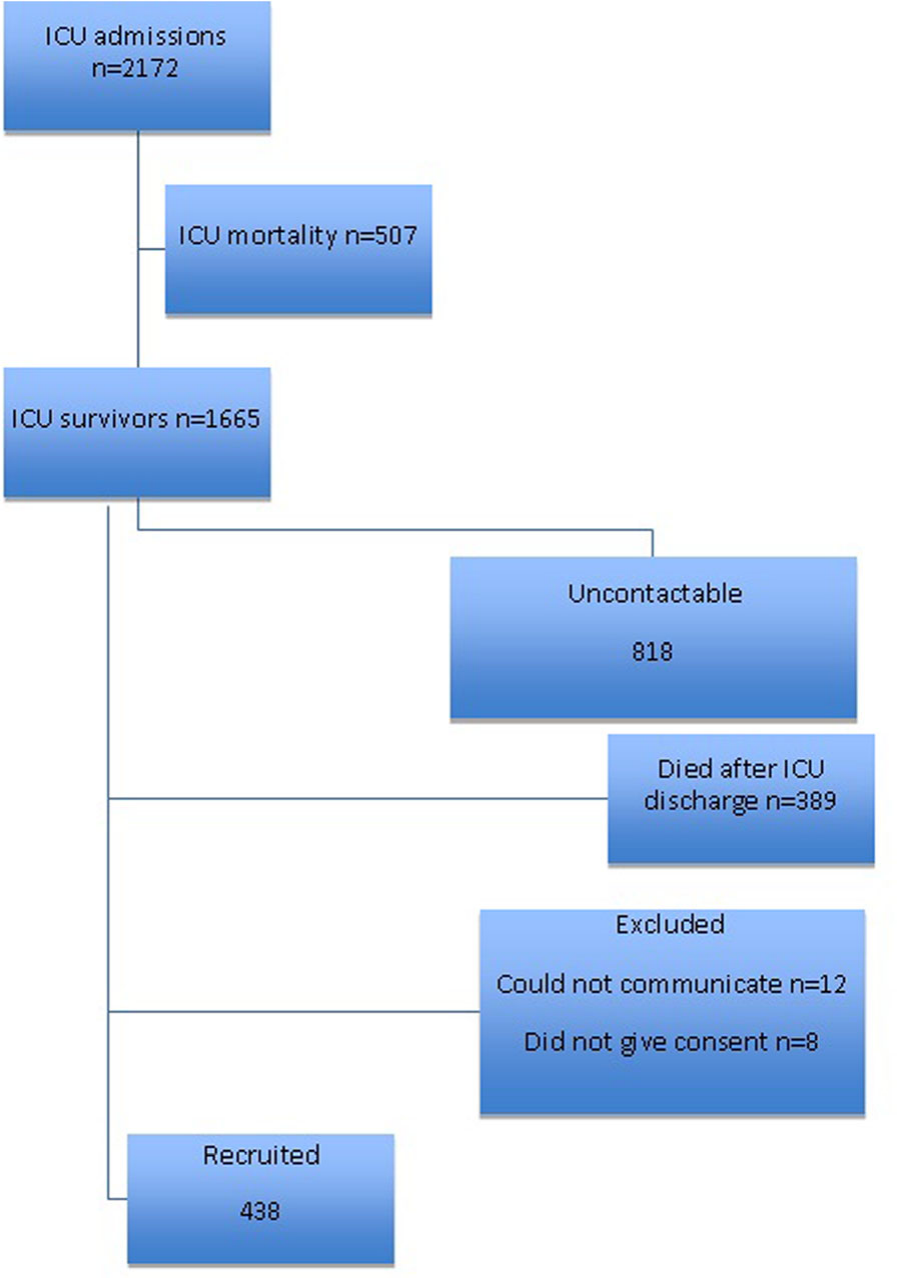
Strobe diagram of the study

Between July and December 2014, 1665 patients were discharged alive from the participating ICU’s. Of these survivors, 818 (49.1%) patients were not contactable and 389 (23.3%) patients died between ICU discharge and day-30 (Fig. 1). Of the 458 (27.5%) survivors contacted, 20 (4.4%) patients were excluded due to inability to communicate 12 (2.6%) or withholding consent 8(1.8%). A total of 438 (26.3%) patients were thus included in the study.

### Admission characteristics

Summary ICU data of surveyed patients is presented in Table 1.

**Table 1:** Summary ICU details of survivors

	No (%) or median (IQR)
Male	207 (47.3)
Median ICU LOS (IQR)	2.0 (3.5)
Mechanical ventilation on admission	104 (24.8)
Vasoactive medication on admission	69 (16.4)
APACHE II Score, Median(IQR)	12 (10)
APACHE II probability, Median(IQR)	0.16 (0.3)

All the continuous variables tested for normality. Age, GCS, LOS, APACHE II score and APACHE II probability were non-normal (*p* = 0.000). The median age was 45.0 (IQR 26.0) years and 48.0% were males. A total of 159 (36.3%) patients were admitted to the ICU for medical reasons, 124 (28.3%) for elective surgery and 95 (21.7%) for emergency surgery. The median (IQR) length of ICU stay was 2.0 (3.5) days and the median (IQR) APACHE II score was 12.0 (10). Mechanical ventilation had been instituted at admission in 107 (24.0%) patients and 69 (16.4%) received vasoactive (vasopressor/inotrope) medication on admission. The most common APACHE II diagnostic categories of those surveyed are shown in Table 2. For 386 (86.4%) patients, this was their first admission to an ICU.

**Table 2:** Common diagnoses of respondents

APACHE II diagnosis	Number(*n* = 438)	Percentage %
Gastrointestinal (surgical)	60	13.7
Bleeding (surgical)	47	10.8
Cardiovascular (non-surgical)	36	8.2
Neoplasm (surgical)	28	6.4
Respiratory (non-surgical)	22	5.0

Patients’ recollection of the ICU experiences is presented in Table 3. Whilst 349 (78.1%) patients were able to remember their admission to the hospital, only 189 (42.3%) could recall their admission into the ICU. The characteristics of the ICU episode for patients who were able to recall their ICU admission are compared with those who were not able to in Table 4. Significant features impacting on patients ability to recall ICU admission include: sedation at admission, mechanical ventilation at admission, administration of vasoactive medications at admission, lower conscious level (GCS) and a higher severity of illness (APACHE II score) (Table 4).

**Table 3:** Consciousness and recollection of ICU admission and stay

Question	Number (% of responses)	
Consciousness		
Do you remember your admission to hospital? Yes	349	78.08%
Do you remember your admission to the Intensive Care Unit (ICU)? Yes	189	42.28%
What do you think regarding the memory of your ICU stay?		
I prefer not to remember	3	0.67%
I don’t remember anything	3	0.67%
I don’t mind to remember	396	88.59%
I want to remember everything	34	7.61%
None of them	11	2.46%
Experiences		
How do you classify your sleep during ICU stay?		
Excessive	112	25.06%
Sufficient and restoring	227	50.78%
Insufficient	108	24.16%
Have you had any dreams during ICU stay? Yes	59	13.20%
If yes how frequently did they occur?		
Daily	25	5.59%
Twice a week	2	0.45%
Once	4	0.91%
Have you had any nightmares during ICU stay? Yes	54	12.08%
If yes how frequently did they occur?		
Daily	11	2.46%
Twice a week	6	1.34%
Once	5	1.12%
3 days	1	0.22%

**Table 4:** Characteristics of patients who recall ICU admission and those who do not

	When admitting to ICU		*p*-value
	Recall (186)	Do not recall (252)	
Mechanical ventilation on admission (%)	25 (14.2)	79 (32.5)	0.000
Sedated on admission (%)	37 (21.9)	114 (51.4)	0.000
Vasoactive medication on admission (%)	20 (11.2)	49 (20.1)	0.015
APACHE II Score median (IQR)	11 (8)	14 (11)	0.000
GCS median (IQR)	15 (0)	15 (6)	0.000
Gender; Male (%)	94 (50.5)	113 (44.8)	0.238
Admission Type (%)			
Emergency surgery	40 (26)	53 (25)	
Medical	66 (42.9)	85 (40.1)	
Planned surgery	48 (31.2)	74 (34.9)	0.751

Three hundred and ninety-six (88.5%) patients surveyed did not object to recalling their ICU experiences (*n* = 396, 88.5%), whereas 3 patients (0.7%) were unable to recall any part of their ICU stay and another 3 patients did not wish to do so.

#### Stress

**Fig. 2:**
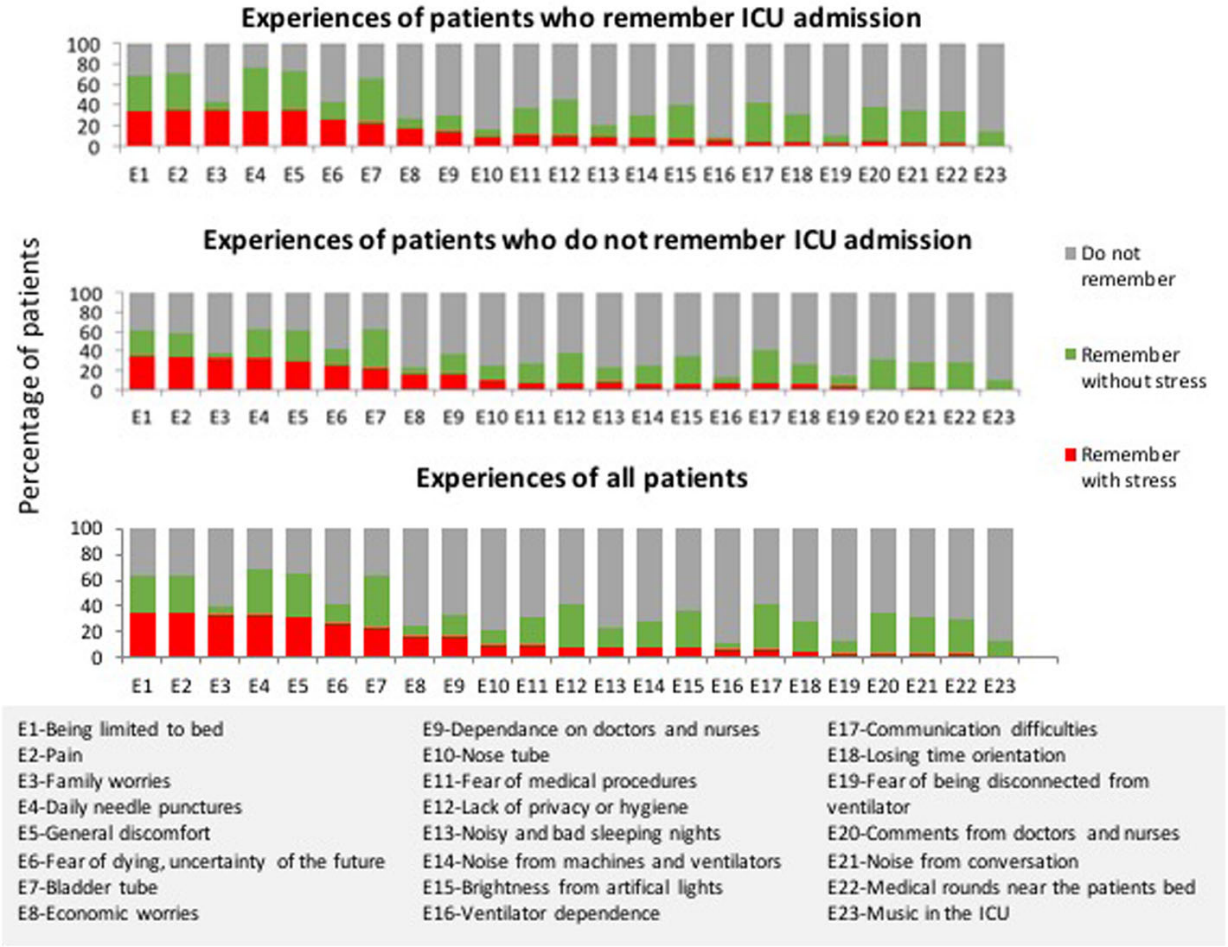
Patients’ recollections of their stressful experiences in the ICU

Patients’ recollections of their stressful experiences in the ICU are shown in Fig. 2. Of the 107 (23.9%) patients who were ventilated at admission, only 53 remembered the experience. Twenty-nine (54.7%) of those who remembered being ventilated found “dependency” on the ventilator to be stressful, and 13 (24.5%) feared being disconnected from it. Confinement to bed, pain and general discomfort, daily blood sampling using needles, family worries, fear of dying or uncertainty in the future were associated with the greatest amount of stress (Fig. 2).

#### Sleep

Two hundred and twenty-seven (50.78%) patients said that their sleep was sufficient and restoring, 112 (25.06%) patients stated that their sleep was excessive and 108 (24.16%) survivors said that it was insufficient. Fifty-nine (13.2%) of the patients recalled dreams during their stay in the ICU and in 25 cases (5.59%) they felt these dreams occurred on a daily basis. Fifty-four patients,(2.8%) stated that they had nightmares with 11 (2.46%) of them reporting that that they experienced these nightmares daily. The majority of patients (376, 84.12%,) found the atmosphere of the ICU to be friendly and calm.

#### Patient satisfaction

**Fig. 3:**
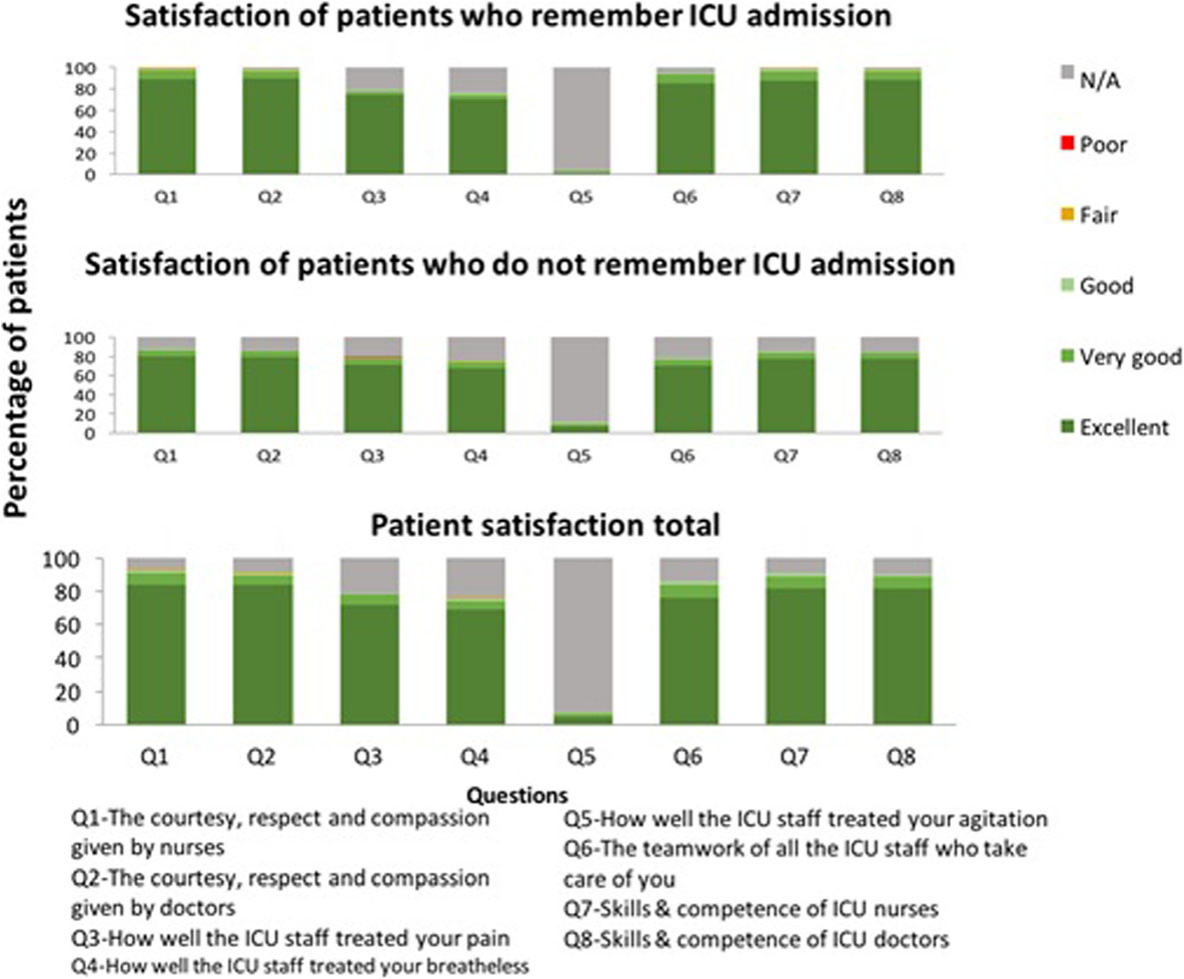
Patients perception on care received

Details of patient satisfaction pertaining to their ICU care are shown in Fig. 3. None of the patients who felt able to comment (91.06%) considered the skills and competence of the doctors and nurses to be poor. Overall, the patients found the level of health care received in the ICU to be “very satisfactory” (*n* = 411, 93.8%) with none stating they were either “dissatisfied” or “very dissatisfied”. Both medical and surgical patients expressed high levels of satisfaction. Patients admitted to medical ICU’s reported the level of health care received in the ICU to be “very satisfactory” (*n* = 190, 95.00%) with none stating they were either “dissatisfied” or “very dissatisfied”. Similarly patients admitted to surgical ICU’s reported their care to be “very satisfactory” (*n* = 221, 92.86%) with none stating they were either “dissatisfied” or “very dissatisfied”.

Three hundred and ninety eight (90.9%) patients made additional comments regarding their carers. These comments included: ICU staff were excellent / very good (*n* = 160), I appreciate the service of ICU staff (*n* = 92), the ICU staff saved my life (*n* = 49), I want to thank them (*n* = 18), the staff were hardworking (*n* = 17), kind (*n* = 15), dedicated to service (*n* = 13) and “God like” (*n* = 9).

#### Recommendations for improvement

Few (*n* = 15) patients made suggestions for improvement; increasing the number of beds in the ICU (*n* = 3), increasing the facilities available to the staff (3), reducing noise in the ICU (*n* = 2), staff (specified by patients as health care assistants) being kinder to patients (*n* = 2), having curtains between patients (*n* = 1), separate cubicles for patients (*n* = 1), increasing the space between beds (*n* = 1), improving technology available in the ICU (*n* = 1) and allowing more visitors in order to reduce stress (*n* = 1) were included in the suggestions.

## Discussion

Patients from thirty-two state ICUs participated in the study, representing differing ICU patient groups, geographical locations and hospital categories. Nearly all patients (91.5%) expressed satisfaction with the ICU care they received and none of the patients surveyed considered ICU nurses or doctors to be lacking in their provision of care.

Inability to recall admission to ICU was relatively common and is in keeping with other studies from LMIC settings, however rate of total amnesia for the duration of the ICU stay was much lower (0.67%) than reported previously [2]. Unsurprisingly, the use of sedation, greater severity of illness and critical care therapies were more prevalent in the group who had limited recall. Details of organ support instituted during the remainder of the ICU stay and details of sedation, analgesia and incidence of delirium were not available, preventing further exploration of possible contributors to amnesia.

Stressful experiences were only experienced by a minority of patients, but the commonest stressful experiences were similar to other settings [2, 21]. Whether the low levels of stressful experiences are due to sample or interviewer bias, lower patient expectations, or cultural, religious or fatalistic views, require further qualitative exploration. In addition, patient satisfaction when evaluated in isolation has been known to be a poor measure of evaluating critical care services in HIC settings [22, 23].

Patient expectations regarding ICU care in Sri Lanka may be different to those of high income countries due to a multitude of factors, including, the fierce competition for scarce critical care services [24–27] relatively low-staffing, overcrowding and low resources in non-critical care wards to which patients are discharged to, and originate from [27, 28], and conversely the relatively well staffed ICU environment [29, 30]. In addition, cultural beliefs regarding critical illness [31] may further influence patient perspectives and need further exploration in this setting [32]. These factors are synonymous with health care structures in other LMIC settings including, but not limited to, Pakistan, Thailand, India, and as such the findings from this study may have relevance beyond Sri Lanka. A next step would be to undertake a further ethnographic exploration of patient experience, public expectations of healthcare alongsidean observational study of patient care delivered in the ICU.

The authors acknowledge the potential bias of the nurse interviewer, and the use of a tool which had not been validated to this setting. The default hierarchical position of authority that health care workers hold in Sri Lanka (a cultural norm in many LMIC settings) needs to be considered. However, the choice of a Sri Lankan clinician and interviewer with expert knowledge of ICU services was a pragmatic choice, and provided opportunity for capacity building within the Sri Lankan nursing profession [33].

### Limitations

49.12% loss to follow up, though significant, was not unique to this study. In addition, 30-day post-ICU mortality was 23.3% of ICU survivors (causes and other details of death were not available), emphasizing the importance of determining post-ICU outcomes. Such information is scare in many LMIC settings [34].

Investment in follow up services and patient advocacy organisations such as ‘patient voices’ (http://www.patientvoices.org.uk/) and the Health experiences research group (http://www.healthtalk.org/) have created a professional and scientifically moderated forum for patients, families and carers to raise awareness of experiences following acute and critical care and to lobby for future healthcare research and quality improvement directives. However, such services are limited in LMIC settings [35]. Our Study, utilising telephone follow up and electronic health information systems (made possible by a critical care registry [34]) offers feasible and resource-efficient opportunities, in a LMIC setting, to access patient experiences and outcomes beyond the hospital door.

As none of the patients expressed dissatisfaction with their ICU care it was not possible to evaluate associations between satisfaction and ICU stressors. A mixed methods evaluation, including quality of life measures, ongoing patient and family experiences, and assessment of PTSD may provide better discriminators when evaluating ICU performance with regard to patient centered outcomes and for exploring the impact of critical care stay on economic and social recovery. A follow- up study in this setting is currently being undertaken by our group.

## Conclusion

In common with HIC, survivors were very satisfied with their ICU care. In contrast to HIC settings, specific ICU experiences were frequently not recalled, but those remembered were reported as relatively stress-free. Stressful experiences, in common with HIC, were most frequently related to uncertainty about the future, dependency, family, and economic concerns.

## Additional file

Additional file 1: Data collection instrument. The data collection instrument contains the original questionnaire. (XLSX 13 kb)

## Abbreviations

APACHE: Acute Physiological and Chronic Health Evaluation
HIC: High Income Country
ICU: Intensive Care Unit
LMIC: Low Middle Income Country
PTSD: Post Traumatic Stress Disorder

## References

[1] Dondorp AM, Iyer SS, Schultz MJ (2016). Critical Care in Resource-Restricted Settings. JAMA.

[2] Granja C, Lopes A, Moreira S, Dias C, Costa-Pereira A, Carneiro A. Patients’ recollections of experiences in the intensive care unit may affect their quality of life. Crit Care. 2005 ;9(2):R96-109. Available from: http://www.pubmedcentral.nih.gov/articlerender.fcgi?artid=1175917&tool=pmcentrez&rendertype=abstract. [cited 2014 Jan 17]. 10.1186/cc3026 PMC1175917 15774056

[3] Khalifezadeh A, Safazadeh S, Mehrabi T, Mansour BA (2011). Reviewing the effect of nursing interventions on delirious patients admitted to intensive care unit of neurosurgery ward in Al-Zahra Hospital, Isfahan University of Medical Sciences. Iran J Nurs Midwifery Res.

[4] Tembo A, Parker V, Higgins I. Being in limbo: The experience of critical illness in intensive care and beyond. Open Journal of Nursing. 2012;2:270–276. Available from: https://www.scirp.org/journal/PaperInformation.aspx?PaperID=23176.

[5] Kralik D, Brown M, Koch T. Women’s experiences of “being diagnosed” with a long-term illness. J Adv Nurs. 2001;33(5):594–602. 10.1046/j.1365-2648.2001.01704.x 11298195

[6] Jones C, Griffiths RD, Humphris G, Skirrow PM (2001). Memory, delusions, and the development of acute posttraumatic stress disorder-related symptoms after intensive care. Crit Care Med.

[7] Jackson J, Ely EW, Morey MC, Anderson VM, Denne LB, Clune J (2012). Cognitive and physical rehabilitation of intensive care unit survivors: Results of the RETURN randomized controlled pilot investigation. Critical Care Medicine.

[8] Hunziker S, McHugh W, Sarnoff-Lee B, Cannistraro S, Ngo L, Marcantonio E (2012). Predictors and correlates of dissatisfaction with intensive care. Crit. Care Med.

[9] Donabedian A (1966). Evaluating the quality of medical care. Milbank Q.

[10] Richard S. Lazarus SF. Stress, Appraisal, and Coping. Springer; 1984.

[11] Stichler JF, Weiss ME (2000). Through the eye of the beholder: multiple perspectives on quality in women’s health care. Qual Manag Health Care.

[12] Wilde-Larsson B, Larsson G, Kvist LJ, Sandin-Bojö AK (2010). Womens’ opinions on intrapartal care: development of a theory-based questionnaire. J Clin Nurs.

[13] Pattison N, Lee M (2014). Two tribes coming together: patient and public involvement in cancer research. Eur J Cancer Care.

[14] De Silva, Haniffa R. A Survey Report on Intensive Care Units of The Government Hospitals in Sri Lanka 2012.

[15] Sahlsten MJM, Larsson IE, Sjöström B, Plos KAE (2009). Nurse strategies for optimising patient participation in nursing care. Scand J Caring Sci.

[16] Wright SE, Walmsley E, Harvey SE, Robinson E, Ferrando-Vivas P, Harrison DA (2015). Family-reported experiences evaluation (FREE) study: a mixed-methods study to evaluate families’ satisfaction with adult critical care services in the NHS. Heal Serv Deliv Res.

[17] Kim LS, Kim GS, Noor Arini I, Nor Zehan A, Salimah J, Rosna AR (2014). Recalling ICU experiences: patients’ perspectives. Middle-East J Sci Res.

[18] McHugh ML (2012). The chi-square test of independence. Biochem Medica.

[19] McKnight PE, Najab J. Mann-Whitney U Test. In: The Corsini Encyclopedia of Psychology 2010.

[20] Royston P (1992). Approximating the Shapiro-Wilk W-test for non-normality. Stat Comput.

[21] Soh KL, Soh KG, Ahmad Z, Ramanm RA, Japar S (2008). Perception of intensive care unit stressors by patients in Malaysian federal territory hospitals. Contemp Nurse.

[22] Sitzia J, Wood N (1997). Patient satisfaction: a review of issues and concepts. Soc Sci Med.

[23] Williams B, Coyle J, Healy D (1998). The meaning of patient satisfaction: an explanation of high reported levels. Soc Sci Med.

[24] Adhikari NKJ, Fowler RA, Bhagwanjee S, Rubenfeld GD (2010). Critical care and the global burden of critical illness in adults. Lancet.

[25] Agyeman-Duah JN, Theurer A, Munthali C, Alide N, Neuhann F. Understanding the barriers to setting up a healthcare quality improvement process in resource-limited settings. BMC Health Serv Res. 2014;14:1. 10.1186/1472-6963-14-1 PMC3880175 24382312

[26] Beane A, De Silva AP, De Silva N, Sujeewa JA, Rathnayake RMD, Sigera PC AP, Mahipala PG, Rashan A, Munasinghe S, Jayasinghe S, Dondorp AM HR. Evaluation of the feasibility and performance existing Early Warning Scores to identify patients at risk for adverse outcomes in Low-Middle Income Country settings. BMJ Open; 2018. 10.1136/bmjopen-2017-019387. 10.1136/bmjopen-2017-019387 PMC5922475 29703852

[27] Beane A, Athapattu P, Dondorp A, Haniffa R. Commentary: challenges and priorities for pediatric critical care clinician–researchers in low- and middle-income countries. Front Pediatr. 2018;6:38. Available from: https://www.frontiersin.org/articles/10.3389/fped.2018.00038/full. 10.3389/fped.2018.00038 PMC5835091 29536985

[28] Aveling E-L, Kayonga Y, Nega A, Dixon-Woods M (2015). Why is patient safety so hard in low-income countries? A qualitative study of healthcare workers’ views in two African hospitals. Glob Health.

[29] Sigera PC, Sanjeewa Tunpattu TMU, Jayashantha TP, De Silva AP, Athapattu PL, Dondorp A (2016). National profile of physical therapists in critical care units of Sri Lanka: lower middle-income country. Phys Ther.

[30] Haniffa R, De Silva AP, Iddagoda S, Batawalage H, De Silva STGR, Mahipala PG, et al. A cross-sectional survey of critical care services in Sri Lanka: A lower middle-income country. J Crit Care. 2014;29(5):764–768. Available from: https://www.ncbi.nlm.nih.gov/pubmed/24929445. 10.1016/j.jcrc.2014.04.021 24929445

[31] Tucker CM, Herman KC, Pedersen TR, Higley B, Montrichard M, Ivery P (2003). Cultural sensitivity in physician-patient relationships: perspectives of an ethnically diverse sample of low-income primary care patients. Med Care.

[32] Khaleghparast S, Joolaee S, Ghanbari B, Maleki M, Peyrovi H, Bahrani NA. Review of visiting policies in intensive care units. Global journal of health science. 2015;8:267–76. Available from: https://www.ncbi.nlm.nih.gov/pmc/articles/PMC4954899/. 10.5539/gjhs.v8n6p267 PMC4954899 26755480

[33] Paskins Z (2001). Sri Lankan health care provision and medical education: a discussion. Postgrad Med J [Internet].

[34] De Silva A, Harischandra P, Beane A, Rathnayaka S, Pimburage R, Wijesiriwardena W, et al. A data platform to improve rabies prevention, Sri Lanka. WHO Bulletin. 2017;95(9):646–651. Available from: https://www.ncbi.nlm.nih.gov/pmc/articles/PMC5578379/. 10.2471/BLT.16.188060 PMC5578379 28867845

[35] Schmidt K, Worrack S, Von Korff M, Davydow D, Brunkhorst F, Ehlert U (2016). Effect of a primary care management intervention on mental health-related qualityof life among survivors of sepsis a randomized clinical trial. JAMA.

